# Plasma cell-free DNA genome-wide methylation profiling enables detection and activity assessment in systemic lupus erythematosus

**DOI:** 10.3389/fimmu.2025.1721954

**Published:** 2025-12-19

**Authors:** Yi-jing Liu, Fang Wang, Yi-qun Zhang, Zhi-hua Pei, Zhen Chen

**Affiliations:** 1 Department of Rheumatology and Immunology, The Second Affiliated Hospital of Fujian Medical University, Quanzhou, Fujian, China; 2 Department of Immunology, Foresea Life Insurance Guangxi Hospital, Nanning, Guangxi, China; 3 School of Life Sciences and Technology, Tongji University, Shanghai, China; 4 Hubei Key Laboratory of Agricultural Bioinformatics, College of Informatics, Huazhong Agricultural University, Wuhan, Hubei, China

**Keywords:** systemic lupus erythematosus, lupus nephritis, cell-free DNA, WGBS, multi-omics

## Abstract

**Introduction:**

Cell-free DNA (cfDNA) has emerged as a promising non-invasive biomarker in precision medicine and is increasingly recognized as relevant to relevance in systemic lupus erythematosus (SLE), where elevated plasma cfDNA levels have been consistently observed. However, the cfDNA methylation pattern in SLE is unclear.

**Methods:**

Differentially expressed genes (DEGs) and differentially methylated probes (DMPs) related to SLE were obtained from the Gene Expression Omnibus (GEO) database. Through multi-omics approaches, the immune microenvironment in renal tissues, whole blood, and peripheral blood mononuclear cells from SLE patients was integrated and analyzed. Applying whole-genome bisulfite sequencing technique, we evaluated the epigenomic features of plasma cfDNA methylation in patients with lupus nephritis (LN). The neural network deep-learning approach was employed to construct a cfDNA-based lupus methylation diagnostic model, which was subsequently validated in two independent cohorts. Finally, the hub genes of the model were screened as potential biomarkers for clinical application in LN patients.

**Results:**

This study found that SLE occurred in abnormal immune microenvironments in multiple sample types, including renal tissue, whole blood, peripheral blood mononuclear cells, and plasma cfDNA, accompanied by abnormal activation of the interferon-related signaling pathway and antiviral response. CfDNA methylation levels were reduced in patients with LN compared to healthy controls, particularly at gene promoter regions. By integrating 13 datasets associated with SLE, and combining them with cfDNA methylation sequencing data, we have screened 163 conserved dysfunctional methylated regions that are related to SLE. The model constructed based on the identified differentially methylated regions demonstrated excellent diagnostic performance for SLE with an area under the curve (AUC) of 0.987, 0.84 in GSE82218 and GSE96879, respectively. Moreover, the model scores differed significantly across disease activity levels (Normal vs. SLE, p<0.0001; low vs. high activity, p<0.01), indicating its potential utility in distinguishing disease states and assessing disease severity.

**Discussion:**

Multi-omics analyses indicate that frequently aberrant methylation sites are correlated with immune-related pathways, disease onset and progression. The analysis of cfDNA methylation profiles can serve as a diagnostic tool for distinguishing disease states and assessing disease severity in SLE.

## 1 Introduction

Systemic lupus erythematosus (SLE) is an autoimmune disorder (1) that could be accompanied by severe complications, such as renal function damage, lupus encephalopathy, and immune thrombocytopenia. Early diagnosis is essential to improve the prognosis (2).

Although several independent small-scale studies have investigated RNA sequencing, gene expression profiling, and methylation microarrays in patients with SLE, these studies demonstrate limited accuracy and reliability (3–5). Multi-omics approaches have achieved substantial progress in oncology, while research integrating diverse sample types and multi-omic analyses in SLE remains limited. With technological progress, the comprehensive analysis based on genome-wide DNA methylation sequencing has expanded the scope of genomic research (6). Methylation level is a dynamic variable that is potentially reversible, which renders it a promising target for both diagnosis and treatment (7). The release of epigenetic information concerning abnormal cells in SLE lesions into the plasma could be detected using liquid biopsy methods, facilitating the diagnosis and monitoring of disease activity. With the recent in-depth study of methylation and SLE, the related mechanism has been gradually clarified. Previous study revealed significant low methylation of interferon (IFN) regulatory genes, including IFI44L, PARP9, and IFITM1 in the CD4+ T cells, B cell, granulocytes and monocytes of patients with SLE (8, 9). Therefore, DNA methylation pattern has profound potential as a biomarker for disease screening and monitoring. Moreover, the related research on plasma cell-free DNA (cfDNA) provides a novel perspective.

CfDNA is the most prevalent analyte in liquid biopsies and represents a highly promising non-invasive biomarker (10). Over the past decade, cfDNA has emerged as a promising biomarker in oncology due to its short half-life and dynamic quantitative changes. A recent cohort study of 350 colorectal cancer patients demonstrated that longitudinal assessment of cfDNA methylation may facilitate early detection of recurrence, thereby improving risk stratification and postoperative management (11). However, the effects of cfDNA on autoimmune diseases are partially understood. Epstein-Barr virus (EBV) has always been suspected to be related to the pathogenesis of SLE. In 2021, Anna et al. found that EB viral load is related to uneven cfDNA fragmentation, elevated cfDNA levels, and kidney disease in patients with SLE (12). In addition, research demonstrated that the plasma cfDNA concentration in patients with SLE was significantly higher than that in healthy individuals and found that the disease medical assessment comprised Systemic Lupus Erythematosus Disease Activity Index (SLEDAI) score, patient symptoms and laboratory parameters showed a significant correlation with the change in cfDNA concentration (13). Although most epigenome-wide studies in SLE describe widespread hypomethylation, the impact of cfDNA methylation levels on SLE pathogenesis remains unclear.

**Figure 1:**
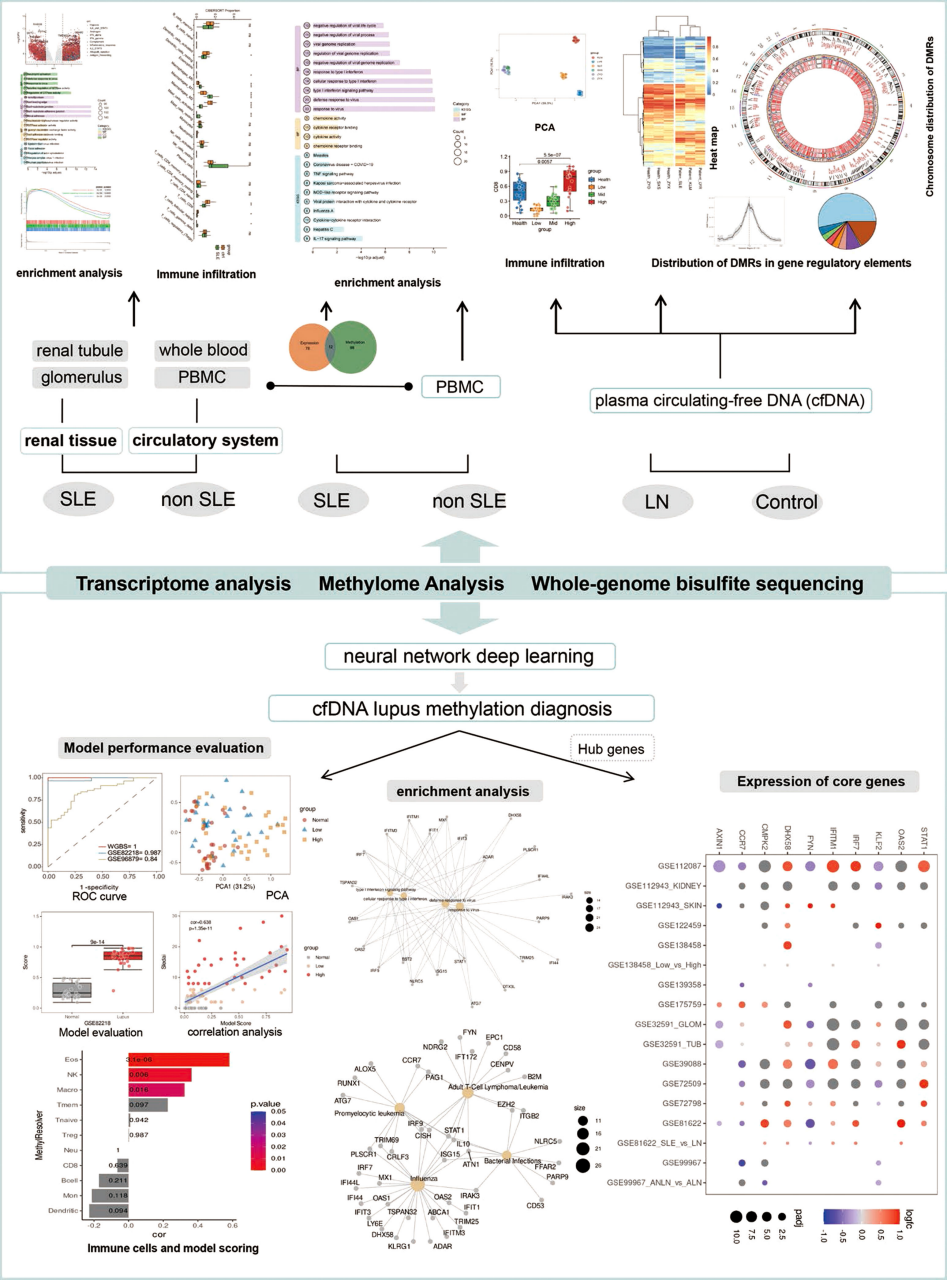
Schematic diagram of the study. This study compared the abnormal immune microenvironment of multiple sample types from SLE patients, including peripheral blood mononuclear cells, plasma cfDNA and kidney tissue, and found that they all had abnormal activation of interferon-related signaling pathways and antiviral responses to varying degrees. For the first time, the methylation landscape of cfDNA in lupus nephritis (LN) patients was analyzed and a multi-omics analysis-based lupus diagnostic model was constructed using neural network deep learning methods, and the model validity was evaluated on an independent validation dataset. Finally, the key immune genes involved in SLE were further screened by using the model.

Our previous study confirmed that cfDNA concentrations in active patients with SLE were significantly higher than those in inactive patients (14). In this study, the methylation landscape of cfDNA in lupus nephritis (LN) patients was analyzed for the first time, and a plasma cfDNA-based lupus diagnostic model was constructed using a deep learning approach with neural networks. The diagnostic model concurrently reflects disease activity in patients. These findings indicate that cfDNA methylation is implicated in the pathogenesis of LN and provide a theoretical basis for the development of liquid biopsy-based diagnostic methods. A schematic of our study design is provided in **Figure 1**.

## 2 Materials and methods

### 2.1 Data collection

We obtained the methylome (HM450 Chip) and transcriptome (RNA-seq or expression array) data of patients with SLE from the Gene Expression Omnibus (GEO) website (https://www.ncbi.nlm.nih.gov/geo/). All analyzed samples were screened using untreated and baseline samples. These included RNA-seq for GSE139358 (LDG/NDN, SLE = 11, Normal=11), GSE122459 (peripheral blood mononuclear cell (PBMC), SLE = 20, Normal=6), GSE72509 (whole blood, SLE = 99, Normal=18), and GSE112087 (whole blood, SLE = 34. Normal=32). GSE138458 (whole blood, SLE = 312, Normal=24), GSE99967 (whole blood, SLE = 42, Normal=17), GSE39088 (whole blood, SLE = 26, N = 42), GSE72798 (whole blood, SLE = 16, Normal=10), GSE81622 (PBMC, SLE = 30, Normal=25) for the expression chip, GSE32591 (Glomeruli/Tubulointerstitium, SLE = 64, Normal=29), GSE112943 (kidney, SLE = 14, Normal=7). GSE82218 (PBMC, SLE = 30, Normal=25), GSE96879 (PBMC, SLE = 57, Normal=33) for methylation chips.

### 2.2 Transcriptome analysis and identification of differentially expressed genes

Expression microarray data were converted from probe IDs to gene symbols using the probe annotation files provided by GEO, and the maximum value of the repeated symbols was selected to represent the expression of this gene. The log2PFKM matrix was used for RNA-seq data, and limma (R package, v3.42.2) was used for differential expression analysis of all transcriptomes. If diseases were grouped with active and inactive LN, both were subjected to differential expression analysis versus normal samples, respectively. DEG screening was performed using p-value<0.05 and top 1,000 to screen upregulated and downregulated significant genes, which were required to be significant in three or more datasets.

### 2.3 Methylation chip analysis and identification of differential methylation genes

Impute (R package, v1.60) was used to pre-process the HM450 microarray data for NA values, and limma was used to evaluate the differential methylation analyses. The differentially methylated probes (DMPs) complied with the following criteria: (i) Absolute value of (treat Mean – control Mean) <= 0.1; (ii) control standard deviation of <0.2; (iii) treat standard deviation of <0.3 (Where Treat is the patient or active group, Control is the healthy or non-active group); (iv) the top 1,000 DMPs of hyper- and hypo-methylation were chosen from each dataset; and (v) the DMPs were significant in more than three datasets. HM450 annotation file was used to assign genes to DMPs, and the matching gene was defined as a DMG.

### 2.4 Whole-genome bisulfite sequencing data processing and analysis

All patients were enrolled according to the 2019 American College of Rheumatology/European League Against Rheumatism SLE classification criteria (15). Patients were excluded if they were gestational or lactating women, or had other diseases, including other autoimmune diseases, tumors, and acute or chronic infections. Any of the following criteria was sufficient for an LN diagnosis (1): active urine sediments (e.g., white cell count of >5/high-power field and exclude urinary infection or red blood cell count of >5/high-power field); (2) a 24-hour quantitative urine protein level of >0.5 g or a urine albumin/creatinine ratio >500 mg/g (50 mg/mmol); (3) evidence of one or more lesions on a renal biopsy based on the International Society of Nephrology/Renal Pathology Society 2003 classification criteria (16). Whole blood samples from three LN patients and three normal controls were collected for WGBS. Following collection into Ethylene Diamine Tetraacetic Acid (EDTA) tubes (BD Vacutainer), peripheral blood from LN patients and healthy donors was subsequently processed within 4 hours of venipuncture. Plasma was collected using a two-step centrifugation method. In the first centrifugation step, approximately 10 mL of whole blood was centrifuged at 4°C (1600g, 10 minutes), and the supernatant plasma was collected. In the second centrifugation step, the plasma obtained from the previous step was placed in a 4°C centrifuge (16,000g, 10 minutes) to collect the supernatant. The final plasma should be stored in a freezer at -80°C until use. CfDNA was isolated from plasma samples (about 4-5ml) using MagMAX cell-free DNA isolation kit (LifeTechnologies, Carlsbad, California, USA). To further evaluate the sample quality, the extracted cfDNA was quantified by Qubit dsDNA HS detection kit (Life Technology Company of Carlsbad, California, USA). All protocols were carried out according to the manufacturer’s instructions. The normal samples were randomly sampled seven times each, and the disease samples were randomly sampled 20 times each. Sampling to a depth of 20 strata resulted in 21 and 60 normal and patient sub-samples, respectively. Raw read clean was evaluated using Fastp (version v0.20), retaining reads of over 50 bp in length, while default parameters were used for the remainder; Bismark (version v0.23.1) was used for genome mapping and CpG site calling; DMP (p<0.01) and differentially methylated region (DMR) (p<0.05) callings were performed using Dispersion Shrinkage for Sequencing data (DSS) (R package, v2.34) after chromosome splitting. For significant differentially methylated sites, the following conditions should be met: (i) the absolute value of the difference between the methylation ratio of DMP in normal and SLE being >0.2; (ii) each DMR covering more than five DMPs. The DMP and DMR positions of WGBS intersect HM450 by Bedtools (version v2.27.1) for gene annotation.

### 2.5 Biological function enrichment and annotation

Functional enrichment analysis was performed using enrichGO and enrichDO of clusterProfiler (R package, v3.14.3) for the DEGs, methylated sites, and corresponding genes. Pathway expression was analyzed using gene set enrichment analysis (GSEA) for upregulation or downregulation of expression in SLE versus normal. Twenty-two immune cells were annotated using CIBERSORT (IOBR, R package, v0.99.9), and immune-related pathway expression levels were calculated using GSVA (R package, v1.34) for Single-sample Gene Set Enrichment Analysis (ssGSEA) scores. These immune-related scores were used to perform an analysis of dynamic differences in pathway or function during disease progression between groups. Methylation immune annotation was performed using MethylResolver (R package, v 0.1.0), and the empirical matrix of kidney_v2_Signature was selected to filter the input methylation matrix of the corresponding locus of WGBS for deconvolution to calculate the proportion of immune cells in the methylome data.

### 2.6 Model building

The steps of model input variable screening were as follows: (i) DEG of SLE vs Normal expression microarray data, and DMG of HM450 were taken to intersect; (ii) the loci corresponding to SLE-specific genes screened by GEO were taken to intersect with DMRs of WGBS data. Finally, 162 DMRs were obtained. GSE82218 and GSE96879 were used as independent validation sets, and the input matrix of the two independent validation sets represented the value of this DMR at this position with the mean of the probes covered by the DMR. The WGBS data were split into the training and the validation sets by 0.6666. The model involved using a deep learning neural network with three hidden layers (neuralnet, R package, v1.44.2) and assessing model performance using the area under the receiver operating characteristic (ROC) curve (AUC) value of the ROC curve.

### 2.7 Statistical analysis

We conducted all statistical analyses with R v3.6.3 (https://www.r-project.org). The difference in scores between the normal and disease groups, including subgroups with varying disease activity, was assessed using the two-sided Mann–Whitney U test. To evaluate the association between discrete variables and clinical groups, we examined them using Fisher’s exact test. The adjusted p-values for DEGs and GSEA were determined by the Benjamini–Hochberg method. Spearman correlation analysis was employed to evaluate the correlation between model score and immunological characteristics. A two-sided p-value of < 0.05 was set as the significance level, and all calculations were based on 95% confidence intervals.

## 3 Results

### 3.1 Changes of immune microenvironment in SLE patients

**Figure 2:**
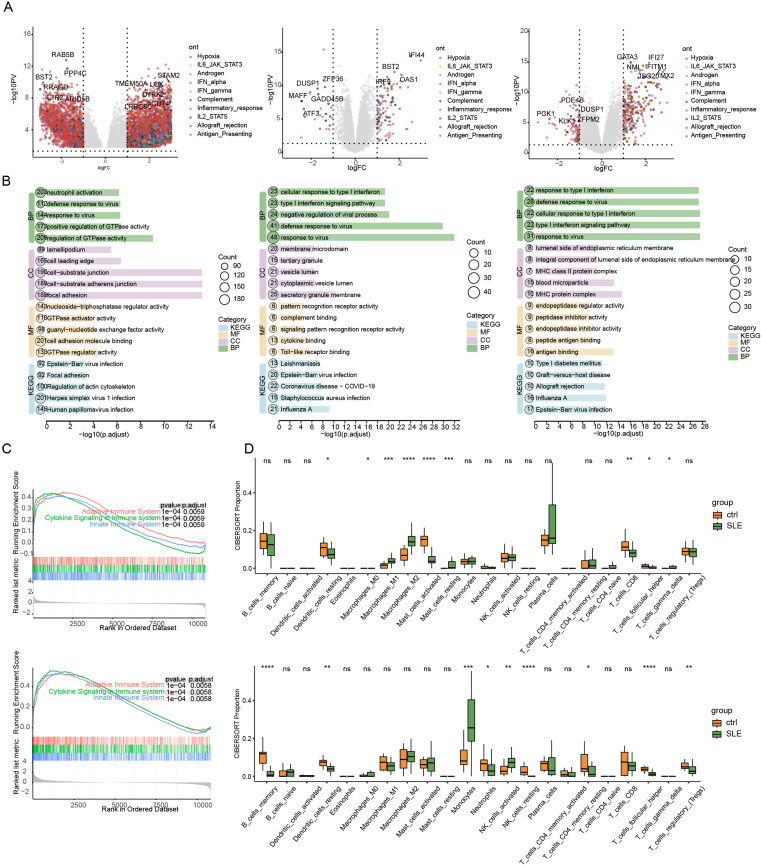
Gene Expression Omnibus database expression profile microarray was used to analyze the immune characteristics of the renal tissue of patients with lupus nephritis (LN). **(A)** The differentially expressed genes between patients with LN and healthy controls in different LN renal tissue-related datasets (from left to right are GSE112943 Tissue, GSE32591 tubules, and GSE32591 glomeruli); **(B)** Enrichment results of Gene Ontology (GO) analysis of upregulated genes in patients with LN (biological process, from left to right are GSE112943 Tissue, GSE32591 tubules, and GSE32591 glomeruli); **(C)** The Gene set enrichment analysis (GSEA) of the tubules(up) and glomeruli(down) in the GSE32591 dataset; **(D)** Cibersort immune-infiltration analysis of the glomeruli(down) and tubules(up) in the GSE32591 dataset. ns, P > 0.05; *P < 0.05; **P < 0.01; ***P < 0.001; ****P < 0.0001.

**Figure 3:**
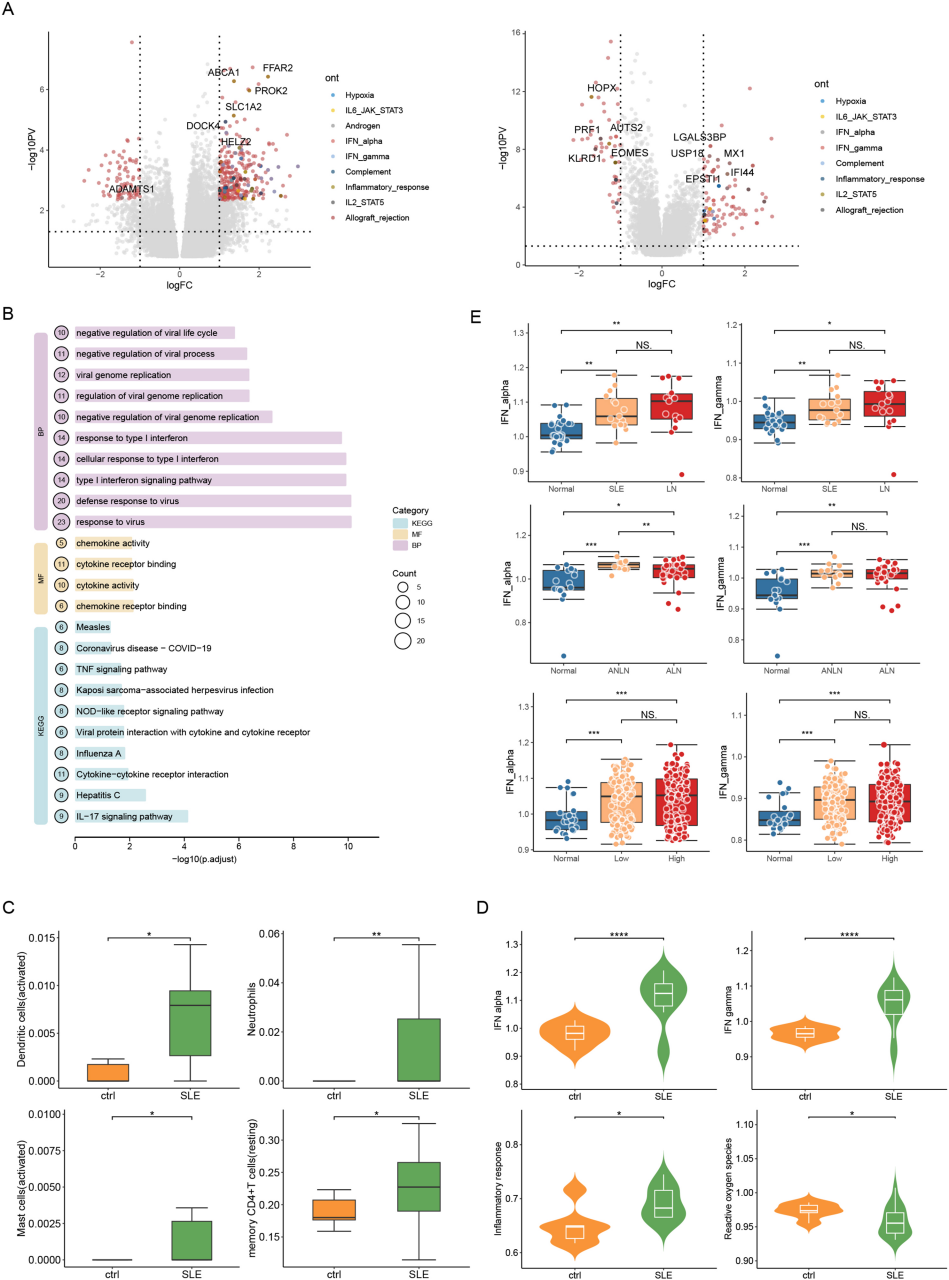
Immune characterization of blood from patients with LN using expression profiling microarrays and RNA sequencing data. **(A)** The Volcano plot of the DEGs between patients with LN and healthy controls. **(B)** Enrichment Analysis of GO and KEGG Pathways of Up-regulated Genes in SLE Patients in the GSE122459 Dataset; **(C)** Immuno-infiltration analysis of peripheral blood PBMC-related dataset GSE122459 in patients with SLE; **(D)** Immuno-microenvironmental analysis of peripheral blood PBMC-related dataset GSE122459 in SLE patients; **(E)** The expression of IFN-α and IFN-γ in healthy controls, patients with SLE, and patients with LN (Top, middle, and bottom are GSE81622, GSE99967, and GSE138458 accordingly). ns, P > 0.05; *P < 0.05; **P < 0.01; ***P < 0.001; ****P < 0.0001.

The aberrant immune activation in SLE drives tissue damage. Conversely, the lesion microenvironment governs the recruitment, differentiation, survival and activation of immune cells, collectively forming the local immune microenvironment. Differentially expressed genes (DEGs) in whole kidney (GSE112943), glomerulus, renal tubule (GSE32591), and peripheral blood mononuclear cells (PBMCs; GSE122459, GSE81622) from patients with SLE were extracted from the Gene Expression Omnibus (GEO) database and matched with immune-related genes. As shown in the volcanic map (**Figures 2A**, **3A**), most immune-related DEGs are enriched in biological processes such as interferon-interferon stimulated genes (ISG), complement, inflammatory response, etc., whether in kidney or PBMCs, and they are important features associated with adverse clinical outcomes in SLE (17, 18). We also found that some genes related to hypoxia pathway also showed abnormal expression, such as CD74, SDC4 and PSEN1. The kidney’s inherent low oxygen tension may be partly responsible. Moreover, LN kidneys present a harsher microenvironment than healthy kidneys (19). The “hardest hit area” of lupus nephritis is the glomerulus. Compared with the volcanic maps of glomerulus and renal tubules from the same cohort, it can also be observed that the abnormal expression of immune-related genes in glomerulus is more than that in renal tubules (**Figure 2A**), but the prognosis of kidney is closely related to the degree of inflammation and injury in renal tubulointerstitial (20). We also noticed that there were some immune-related DEGs in renal tubules similar to glomeruli. Renal tubular epithelial cells can produce inflammatory cytokines (such as IL-6, CX3CL1, TNF-α, IL-1b) (21) similar to glomerular intrinsic cells, which cannot be ignored.

**Figure 4:**
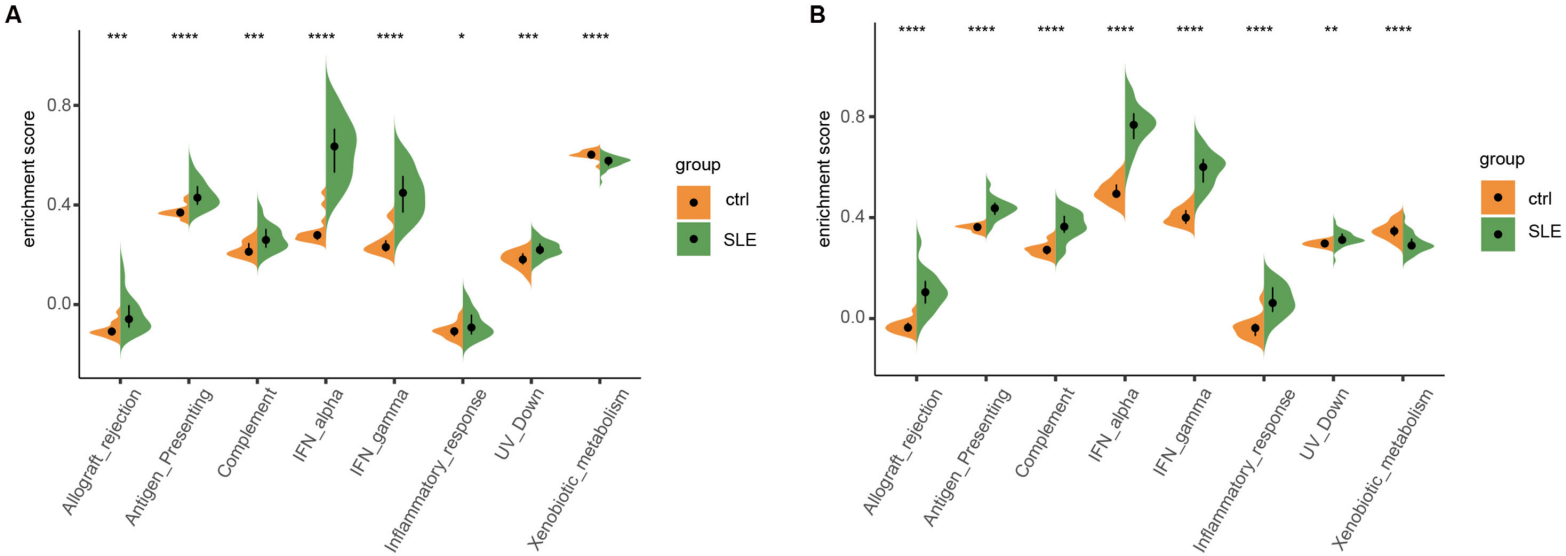
Immuno-microenvironmental analysis of tubules and glomeruli in patients with LN.Immuno-microenvironmental analysis of the tubules **(A)** and glomeruli **(B)** in patients with LN using dataset GSE32591, including allograft, antigen, complement, interferon (IFN)-α, inflammatory response, ultraviolet ray (UV), Xenobiotic metabolism, and IFN-γ. left: tubules, right: glomeruli; ns, P > 0.05; *P < 0.05; P < 0.01; P < 0.001; ***P < 0.0001.

Cibersort algorithm was employed to determine the immune characteristics of SLE. The distribution of abundances for 22 immune cell types was displayed using Vioplot. Mononuclear macrophages were markedly infiltrated in the glomeruli (p<0.05) and tubules (p<0.05) in patients with LN (**Figure 2D**). Macrophages play a very complicated role in the pathogenesis of LN. They not only participate in antigen presentation and immune complex clearance, but also modulate inflammatory responses and tissue restoration through different biological pathways (22). In addition, the number of regulatory T cells (Treg) in LN glomeruli was significantly lower than that in normal glomeruli (P < 0.01) (**Figure 2D**), and Treg was essential for maintaining autoimmune tolerance (23). The reduction in the number or function of Treg cells is related to the pathogenesis of various autoimmune diseases (23). On the other hand, plasma cells infiltrated obviously in LN renal tubules (**Figure 2D**). Plasma cells in B cells promote autoimmunity by producing autoantibodies, which leads to the formation of immune complexes (24). Belimumab, an inhibitor of B cell activating factor, and Rituximab, a monoclonal antibody targeting Cd20 antigen, have been used to manage systemic lupus (25). The distribution of 28 kinds of immune cells provided by ssGSEA analysis also confirmed the above findings (**Supplementary Figure S1B**). The relationship between local tissue and immune status was analyzed through the immune microenvironment, and inflammatory response, complement, IFN-a, and IFN- γ were significantly active (**Figure 4**). These results suggest that LN causes an abnormal renal immune microenvironment (whether in the glomeruli or renal tubules), primarily characterized by the abnormal activity of IFN-related signaling pathway and antiviral response and significant infiltration of the mononuclear macrophage system.

In whole blood transcriptome chips GSE39088, GSE72509, GSE72798, GSE112087 and GSE138458, it was found that compared with healthy people, monocytes, macrophages, activated dendritic cells and plasma cells in SLE patients increased significantly, while resting NK cells decreased (**Supplementary Figure S2**). Plasmacytoid dendritic cells (the main IFN-producing cells) accumulate in the glomerulus of patients with active LN (26), and the secreted IFN-a further stimulates B cells to differentiate into antibody-producing plasma cells (18). PBMCs can better mimic the *in vitro* blood immune environment than whole blood samples do because PBMCs remove multinucleated cells and erythrocytes and primarily contain lymphocytes, monocytes, phagocytes, dendritic cells, and a few other cell types (27). We found that the levels of activated dendritic cells and mast cells in PBMCs of SLE patients increased (**Figure 3C**). However, different from the immune characteristics of renal tissue, the results of Cibersort showed that the abnormal infiltration of neutrophils in PBMCs of SLE patients was more prominent (**Figure 3C**). The release of neutrophil extracellular traps (NETs) is one of the first defense lines of neutrophil against bacteria, viruses, protozoa and other pathogens. The failure of NETs clearance is especially considered as the trigger of renal lesion (LN) in SLE patients (28, 29). To sum up, autoantibodies attack renal tissue of SLE patients, which leads to the enhancement of immune microenvironment in renal tissue under inflammatory stimulation, and blood, as one of the circulating media of immune cells, also shows inflammatory changes. However, the changed immune cell types are slightly different in blood and renal tissue, which may be related to the complex immune infiltration mechanism.

The Kyoto Encyclopaedia of Genes and Genome (KEGG) pathway and gene ontology (GO) analyses of DEGs showed that “response to virus” and “Type I IFN signaling pathway” were profoundly active in the glomeruli and renal tubules (false discovery rate (FDR<0.05) (**Figure 2B**), while the expression of metabolism-related pathways was decreased (FDR<0.05) (**Supplementary Figure S1A**). To make up for the possible omissions and errors caused by the simple use of DEG analysis, we conducted the GSEA of all genes. The results revealed that the genes in the glomeruli and renal tubules of patients with SLE were enriched in the pathways related to innate and acquired immunity (**Figure 2C**), suggesting the significance of abnormal immune microenvironment in the occurrence and development of LN. Enrichment analysis also confirmed that the immune activity of PBMCs in patients with SLE primarily included activation of IFN-related pathways and viral response. In addition, the expressions of the IL-17 (has04657, p<0.001) and nod-like receptor (has04621, p<0.05) signaling pathways were enriched in PBMCs (**Figure 3B**, **Supplementary Tables S1**, **S2**). IL-17 and NOD-like receptor signaling pathways are associated with inflammation (30).

In a word, the immune system abnormalities in the blood and kidney microenvironment of SLE patients mainly include interferon-related signal pathways, antiviral reactions and other immune inflammatory reactions. The results of blood immune microenvironment analysis are also highly consistent with those of renal tissue immune microenvironment analysis of LN patients (**Figure 3D**, **Supplementary Figure S3**). In addition, we found that the level of IFN-α in patients with lupus nephritis is generally higher than that in SLE patients (**Figure 3E**).

### 3.2 PBMC methylation alterations reveal signatures of aberrant immune hyperactivation in SLE

Transcriptional regulation is influenced by epistasis, so we continue to analyze the methylation difference between SLE patients and healthy people. Differential methylation regions (DMRs) were detected from methylation chip (GSE96879) and mapped to the corresponding genes, namely differential methylation genes (DMGs). DMG enrichment analysis demonstrated that the hypermethylated gene of PBMCs in patients with SLE was enriched in “natural killer cell proliferation”, “adipocytokine signaling pathway”, “AMPK signaling pathway”, and other activities. Hypomethylated genes were enriched in processes, such as “Type I IFN signaling pathway”, “response to virus”, and “negative regulation of viral life cycle”. This suggests an association between DNA methylation and aberrant expression of immune-related genes in patients with SLE (**Supplementary Table S3**).

**Figure 5:**
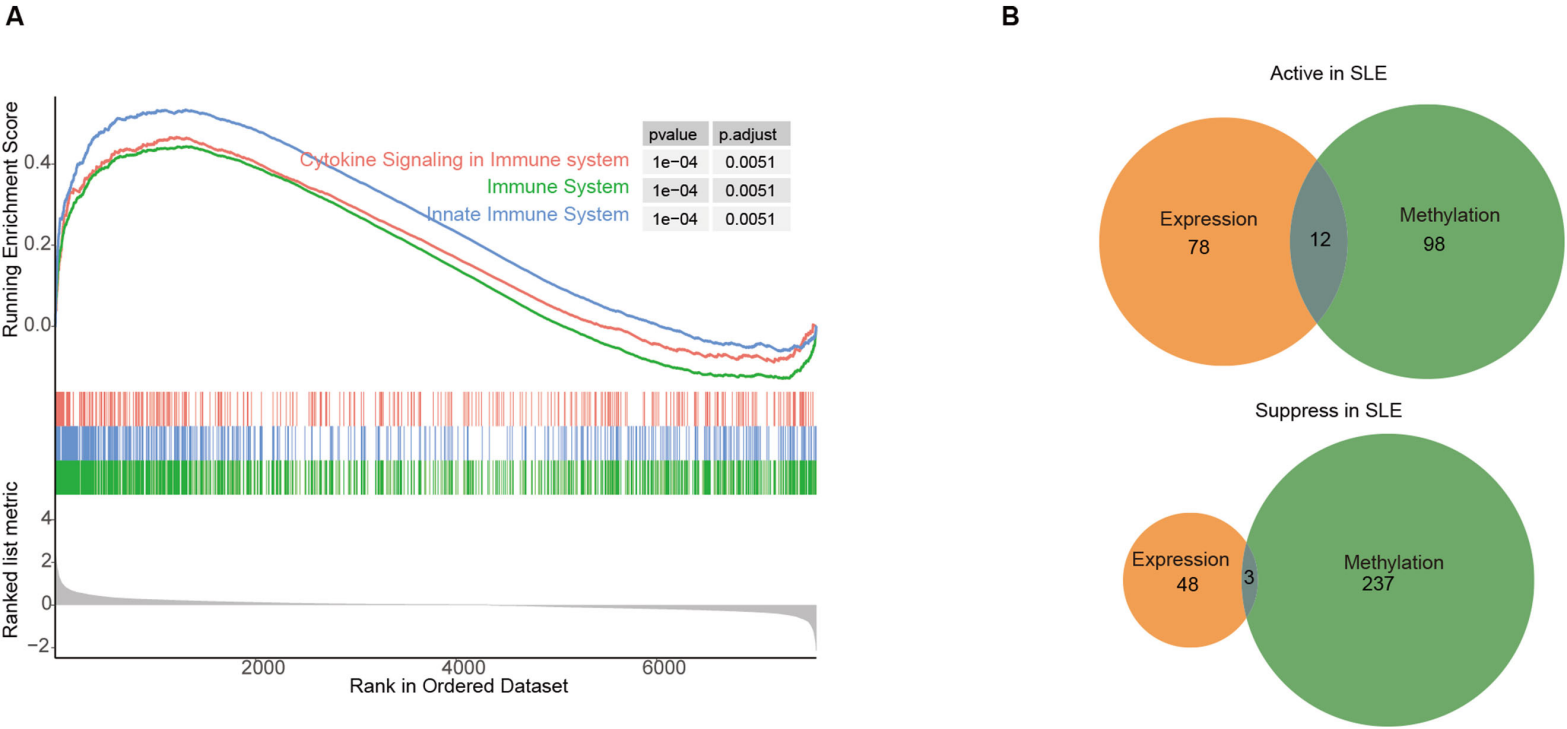
Multigroup analysis of PBMCs in the peripheral blood of patients with SLE. **(A)** GSEA analysis of peripheral blood mononuclear cell (PBMC) expression profile microarray GSE81622 in patients with SLE; **(B)** The PBMCs of patients with SLE were also GSE81622 expression profiling microarray and GSE82218 methylation microarray performed, and hypermethylated downregulated genes and hypomethylated upregulated genes were selected and merged.

To understand the relationship between gene methylation and transcription in SLE patients, we compared the differential analysis of GSE82218 methylation and GSE81622 transcriptome chips from the same SLE PBMCs cohort. For DMG functional pathway enrichment analysis of the DNA methylation chip (GSE82218), the highly methylated genes were primarily enriched in T cell activation and NK cell-mediated cytotoxicity, which was consistent with the enrichment analysis of downregulated genes in the transcriptome chip (GSE81622). The hypomethylated genes were largely enriched in the Type I IFN-related signaling pathway, viral response, antiviral response, and other processes, which was consistent with the enrichment analysis of upregulated genes in same present the transcripome chip (**Supplementary Figure S4**). The GSEA of the GSE81622 dataset also showed high expression of genes related to “Cytokine Signalling in the immune system”, “immune system”, and “innate immune system” in patients with SLE (**Figure 5A**). This implies that DNA methylation patterns can evidently reflect the immune status of patients with SLE, emphasizing that the regulation of DNA methylation on gene expression level is involved in the occurrence and development of SLE.

To screen the conserved methylated genes involved in the SLE process, we focused on the combination of “hypermethylated genes with downregulated expression” and “hypomethylated genes with upregulated expression”. There were three hypermethylated downregulated genes, namely PRF1, PYHIN1, and CD160, and 12 hypomethylated upregulated genes, namely IFI27, LGALS3BP, USP18, MX1, IFI44, IFI44L, IFITM3, HERC5, RSAD2, IFIT1, OAS3, and IFIT3 (**Figure 5B**). The 15 genes were analyzed using Metascape online analysis tool (31), and these genes were primarily enriched in biological processes, such as response to virus (GO:0009615), innate immune response (GO:0045087), antiviral innate immune response (GO:0140374), and response to Type I IFN (GO:0034340). It is suggested that PBMC aberrant methylation and transcript levels are involved in the immune aberrant program of SLE.

### 3.3 Genome-wide cfDNA methylation analysis revealed extensive epigenetic signatures associated with LN

**Figure 6:**
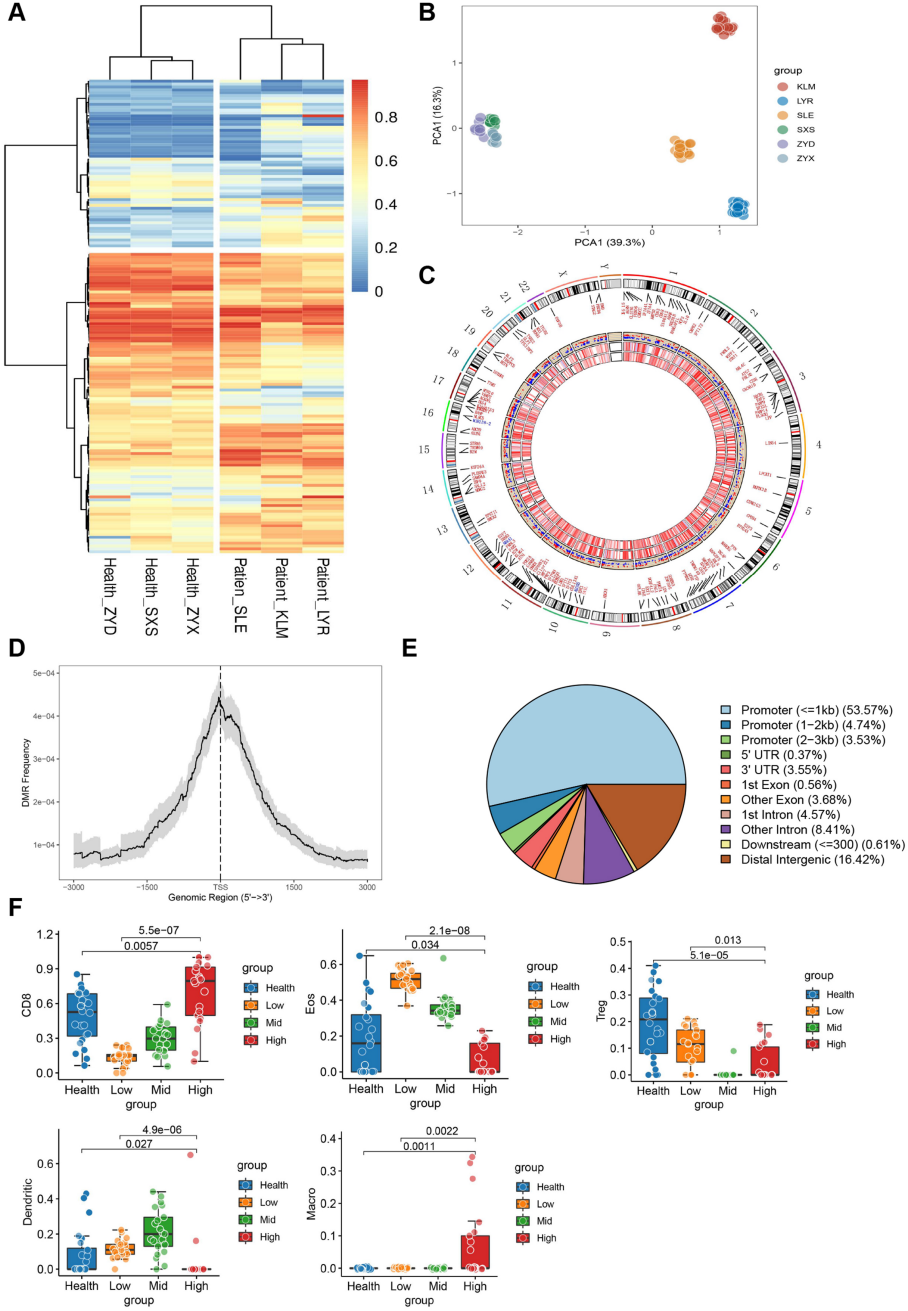
Spatial landscape of whole genome methylation sequencing of plasma cell-free DNA (cfDNA) in patients with LN and controls. **(A)** Cluster analysis of genome-wide methylation profiles of plasma cfDNA in patients with LN and controls; **(B)** Principal component analysis of the methylation profiles of different groups; **(C)** Chromosomal location information for differentially methylated regions (DMRs)/differentially methylated genes (DMGs) of plasma cfDNA from patients with LN (In the scatter layer, red represents SLE hypermethylation and blue represents SLE hypomethylation.); **(D)** Most of the DMRs of plasma cfDNA from patients with LN are located within 200 kb of the transcription start sites of the corresponding genes; **(E)** Distribution of DMRs of plasma cfDNA in gene regulatory elements in patients with LN; **(F)** Bar graphs for Healthy, Low, Mid, and High show immune cell levels in controls and mild, moderate, and reconstituted levels of disease activity.

Our previous studies confirmed that the serum cfDNA concentration in active SLE is significantly higher than that in non-active SLE (14); however, the effect of cfDNA methylation level on SLE remains poorly understood. Therefore, we performed whole genome methylation sequencing using plasma cfDNA from patients with LN and healthy controls. To ensure data reproducibility within the group, principal component analysis was used to identify methylation characteristics (**Figure 6B**). Different component clusters were observed in each group, and a robust distinction was observed between SLE and healthy controls. Therefore, these DMRs can be exploited for subsequent cfDNA methylation analyses and the construction of SLE diagnostic models. The different methylation patterns of LN patients and healthy controls are shown in the heat map (**Figure 6A**). On the whole, the methylation levels of plasma cfDNA were significantly lower in LN patients than in healthy control. 5,384 DMRs were identified in patients with LN and healthy controls, including 3,143 and 2,241 hypomethylated and hypermethylated regions, respectively. The visual DMR signals of hypomethylated and hypermethylated mapped to the entire genome are shown in **Figure 6C**.

DNA methylation functions differently when it occurs in different regions. Gene expression is repressed when gene promoters and transcription start sites (TSS) are hypermethylated, while gene expression is promoted when genosomes are hypermethylated (32). Most of the DMRs of plasma cfDNA in patients with LN were within 200 kb of the TSS of the corresponding gene (**Figure 6D**). Visualization of DMR distribution in gene regulatory elements showed that a considerable part of the DMRs was in the promoter region (**Figure 6E**). This indicates that cfDNA methylation in patients with SLE negatively regulates gene transcription and affects the occurrence and progression of subsequent diseases.

Furthermore, the potential biological functions of the DMGs in cfDNA were evaluated. The cfDNA DMR enrichment analysis was highly consistent with the renal tissue DEG enrichment analysis (**Supplementary Figure S5**). Hypermethylated regions were enriched in biological processes, such as “regulation of cell morphogenesis” and “regulation of axonogenesis”, while genes with reduced methylation levels were primarily related to the “regulation of GTPase activity”, “EB virus infection”, “cellular response to Type I IFN”, “Type I IFN signaling pathway”, and other life activities. These results further support that cfDNA methylation changes in patients with LN reflect the immune status of the LN kidney tissue and are related to the immune response in these patients.

Evaluating the immune characteristics of cfDNA, as shown in **Figure 6F**, the general decrease in T-regulatory cells in patients with SLE was involved in the onset and progression of organ inflammation in SLE (33), while monocyte infiltration was significant, which is similar to the pattern of immune infiltration in the renal tissue of LN patients (**Figure 2D**). According to the disease activity grouping, we also noted that in patients diagnosed with LN, the levels of CD8 cell and neutrophil infiltrations were positively and negatively associated with the disease severity, respectively. Dendritic cells level was increased significantly in patients with mild to moderate active LN, but decreased in patients with high disease activity. The results suggest that detecting the distribution of immune infiltration in peripheral blood can be used to evaluate the disease activity of LN and dynamically predict the disease severity.

### 3.4 Conserved immune-related loci accurately predict SLE

We used the GEO database of SLE-related DEGs, differential methylation sites, and our cfDNA whole genome methylation sequencing results to take the concatenation-generated 163 SLE-related DMRs as the input variables of the model and used neural network for deep learning to build the cfDNA lupus methylation diagnosis (CFLMD) (**Supplementary Table S4**, **Supplementary Figure S6**).

**Figure 7:**
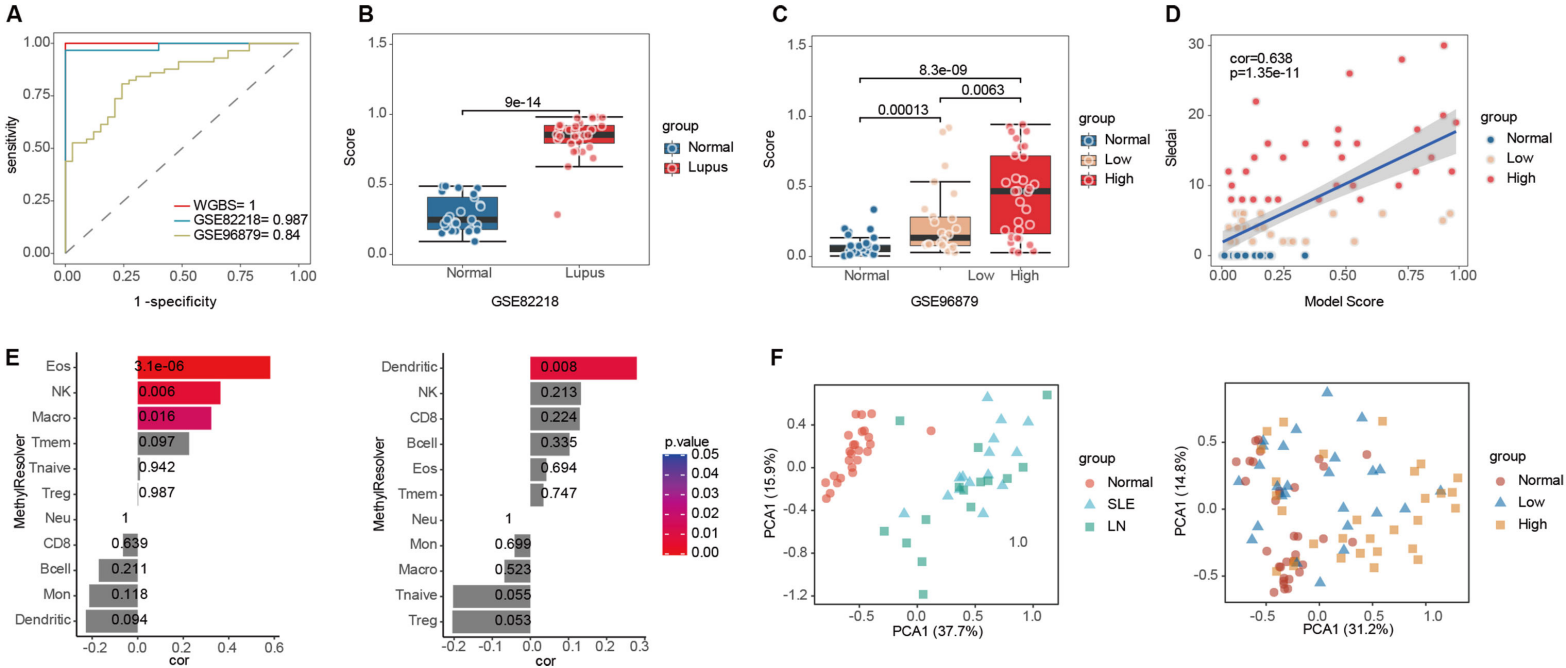
Efficiency validation of the SLE diagnostic model. **(A)** The receiver operating characteristic (ROC) curve of the model in two verification sets; **(B)** Validating diagnostic performance with dataset GSE82218 and model scores; **(C)** Validating diagnostic performance with dataset GSE96879 and model scores; **(D)** The model score is related to the Systemic Lupus Erythematosus Disease Activity Index score in the GSE96879 dataset; **(E)** Relationships between immune cells and model scores were calculated using MethylResolver in the GSE82218(left) and GSE96879(right) datasets; **(F)** Principal component analysis of two independent datasets (left: GSE82218, right: GSE96879) based on the DMR of the model.

Two independent verification data sets are applied in the assessment of the model’s accuracy, and the validity of the model is measured by the area under ROC curve (AUC) of the verification data. We found that, compared with the WGBS, the model had an AUC of 0.987 in GSE82218 and 0.84 in GSE96879 (**Figure 7A**). The model showed good detection performance in both verification sets. Principal component analysis was performed on two independent datasets (GSE82218, GSE96879) according to the model DMRs. As shown in **Figure 7F**, SLE patients can be clearly distinguished from normal participants. These data show that the diagnostic model based on plasma cfDNA methylation serves as a robust, non-invasive method for detecting SLE. To further assess the model’s resilience, the model was independently evaluated in the dataset GSE82218, and the healthy participants and patients with SLE could be distinguished accurately according to the model score (**Figure 7B**). Furthermore, in the dataset GSE96879, the model score could distinguish between patients with mild or severe disease activity (**Figure 7C**). Moreover, the correlation analysis using the GSE96879 dataset showed a strong correlation between model score and disease activity, and the correlation coefficient reached 0.638 (p<0.001) (**Figure 7D**). In conclusion, the CFLMD can be employed to screen SLE patients accurately, and the model scores reflect disease activity notably. Evaluating the relationship between immune cells and model scores using MethylResolver revealed that mononuclear macrophages and dendritic cells were strongly positively correlated with lupus model scores (**Figure 7E**), which was consistent with the trend observed in peripheral blood. Notably, higher eosinophil counts were aligned with increased lupus model scores. Current studies on eosinophils have focused on helminth infections (34) and pathogenic effects on allergic diseases (35); however, eosinophils reportedly modulate inflammatory responses (36). Our results suggest that eosinophils play a role in the disease progression of LN and are potentially associated with active renal lesions.

### 3.5 Conserved lupus-related hub genes are correlated with immune homeostasis

**Figure 8:**
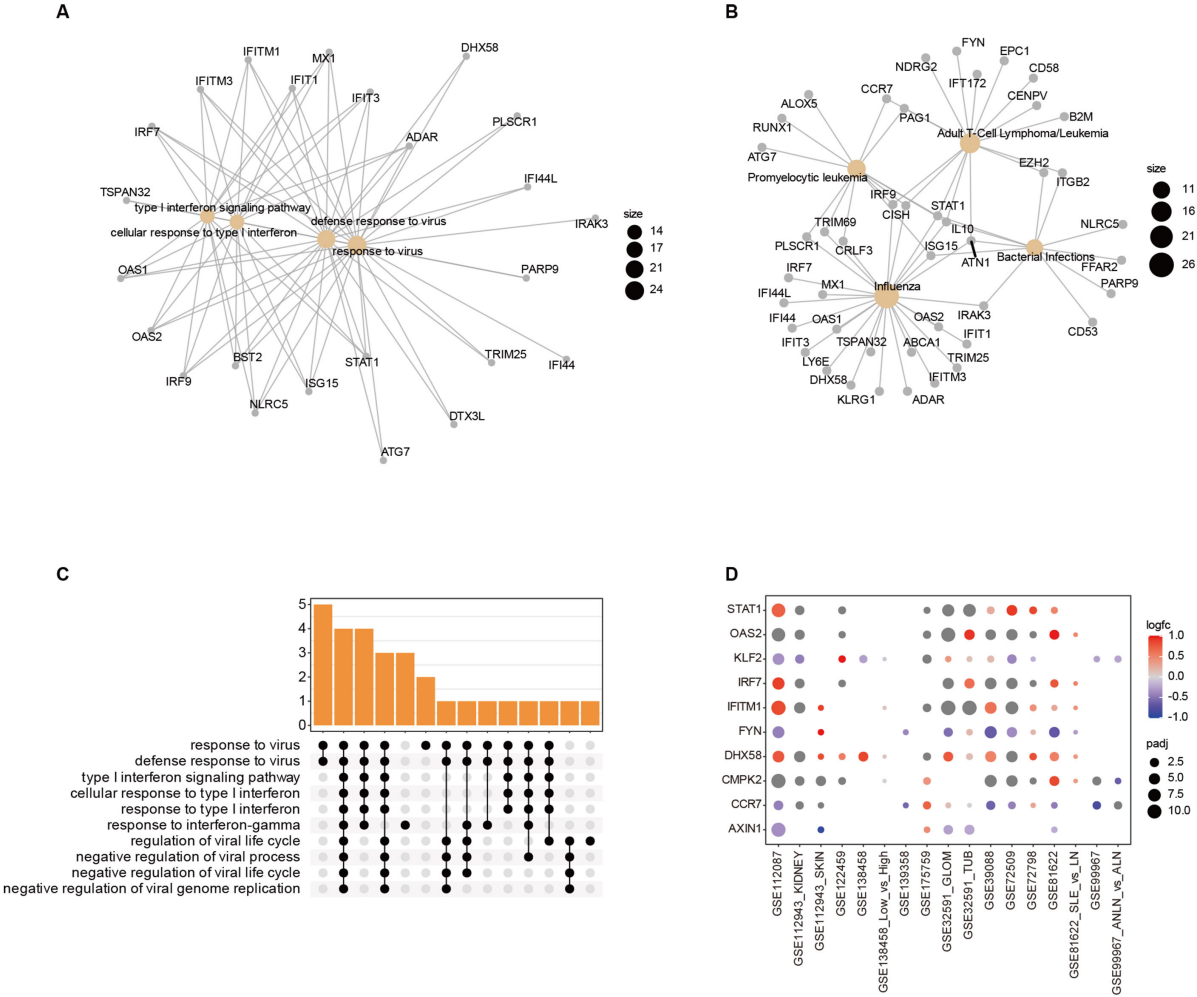
Immunological characterization of the model hub gene. **(A, B)** The GO analysis of model genes; **(C)** The DO analysis of model genes **(D)**. The relative expression of model Hub genes in SLE-related datasets.

Although the remaining input variables of the model were little after the intersection, they could be enriched into immune-related activities, such as antiviral response and Type I IFN signaling pathways (**Figures 8A, C**). The disease Ontology (DO) is employed to investigate the underlying associations between genomics and human diseases. To improve interoperability, the Disease Ontology integrates formal definitions from other ontologies, which are used to describe disease characteristics and causes (37). The DO analysis revealed that the DMR of the model was highly involved in infection and hematological diseases, such as bacterial infection, viral infection, and many leukemia forms, such as promyelocytic leukemia and adult T-Cell lymphoma/leukemia. The enrichment results and their specific association with genes were visualized using cnetplot (**Figure 8B**). This suggests that there is an important association network between SLE and infections and hematological diseases.

The model hub genes included KLF2, IRF7, DHX58, STAT1, FYN, OAS2, IFITM1, CCR7, AXIN1, and CMPK2, most of which are key genes related to IFN, such as IRF7, OAS2, STAT1, IFITM1, DHX58, CCR7, KLF2, and FYN. In addition, IRF7, OAS2, STAT1, IFITM1, and DHX58 are primarily involved in biological processes related to antiviral response. CCR7, OAS2, DHX58, and CMPK2 are involved in response to bacteria, and AXIN1 is primarily involved in the positive regulation of protein phosphorylation (38). To evaluate the expression of these hub genes in several datasets (**Figure 8D**), the expression level of each gene is relatively stable, which is the basis for the robust performance of the model.

## 4 Discussion

The objective of this study was to assess the similarities and differences in the immune microenvironment of different samples from patients with SLE, using multi-omics to depict, for the first time, the landscape of generalized hypomethylation of plasma cfDNA in patients with LN and develop an accurate and reliable diagnostic model based on cfDNA methylation, ensuring that our model responds to disease activity in SLE in a non-invasive manner.

Previous studies have preliminarily observed abnormal changes in transcriptomics and epigenomics of SLE patients (39), and confirmed that the change of epigenetic pattern is one of the signs of SLE immune cells. Our results proved that the immune microenvironment in the renal tissue of patients with SLE was highly consistent with that in blood: there are significant infiltration of mononuclear macrophages and plasma cells. Enrichment analysis further demonstrated the regulatory effect of methylation on DEGs in SLE, particularly in IFN-related signaling pathways and antiviral responses. A recent study conducted at Stanford University has revealed that Epstein-Barr virus (EBV) is a direct inciting factor for SLE. They developed an EBV-specific single-cell RNA-sequencing platform and which substantiates EBV’s mechanism of action by infecting and reprogramming nuclear antigen-reactive B cells, transforming them into highly activated antigen-presenting cells with the potential to drive systemic autoimmune responses, thereby providing a mechanistic foundation for EBV as a driver of SLE (40).We have found that the EBV-related pathway is abnormally highly expressed, with significant changes in the methylation of related genes, which provides a theoretical basis for subsequent research and clinical research treatment drugs.

SLE is a disease with multi-system involvement. Most of the reported multi-omics analyses related to SLE have a single sample type, which leads to the results that cannot fully explain the pathological mechanism of SLE. Some researchers sought to explore the significance of plasma cfDNA in SLE (13, 14);however, in most studies, a significant correlation has not been identified between cfDNA levels and SLEDAI (41, 42). We assessed the spatial landscape of plasma cfDNA methylation in LN patients for the first time, analyzed the SLE immune microenvironment based on differentially methylated regions of plasma cfDNA, such as a decrease in regulatory T cells (Treg), significant infiltration of monocytes, and identified a wide range of LN blood markers. The CfDNA DMR enrichment analysis revealed results that were highly consistent with those of renal DEGs in patients with SLE. In addition, the correlation between the infiltration of CD8 cells and eosinophils and LN severity was found by analyzing the immune characteristics of cfDNA. Liquid biopsy based on cfDNA methylation is a feasible research direction for evaluating the disease activity of LN.

Multi-omics characterization is advantageous for the accurate diagnosis of disease, especially in the detection of disease activity. Simple IFN-related biomarkers have been adopted in several studies to diagnose and evaluate the recurrence and remission of SLE; however, their diagnostic efficiency remains controversial (43, 44). It is considered that the high heterogeneity of SLE, the limited sample size in these studies, or the limitations of histology limited to a single level affect the diagnostic efficiency of these biomarkers (45). In our analyses, the combination of cfDNA methylation profiling and transcriptomics/methylomics of renal and peripheral blood samples for the diagnosis of SLE yielded excellent diagnostic results in multiple cohorts, and model scores showed a strong correlation with disease activity.

CfDNA are the DNA molecules that are released into the tissue fluid from cells all over the whole body (46). encompassing both mitochondrial DNA (mtDNA) and nuclear DNA (nucDNA) (46). Reduced levels of mtDNA correlate with increased mitochondrial defects (47). In summary, Studies have already demonstrated that, SLE patients tended to have lower mtDNA copies and higher nDNA copies in their plasma cfDNA (46). In another study, it was reported that the mtDNA/nucDNA ratio is lower in the whole blood of patients with SLE compared with age matched controls, indicating increased mitochondrial dysfunction in SLE, among patients with SLE with renal involvement, the mtDNA/nucDNA ratio was further reduced (48). It is generally believed that multiple abnormalities of mitochondria, including functional changes, oxidative stress, genetic polymorphisms, mitochondrial DNA (mtDNA) mutations, and apoptotic pathways, are closely related to the pathogenesis of lupus, particularly oxidative stress (49). Oxidative stress leads to impaired mitochondrial function, which in turn produces reactive oxygen species and triggers autoantigenicity and proinflammatory cytokines, leading to SLE (50). Unfortunately, this study did not detect nuclear or mitochondrial DNA-coding genes that regulate mitochondrial dysfunction in SLE, and the key biological processes or genes we screened did not focus on metabolism or metabolic stress. Potential reasons for this consideration include: Our analysis included Whole-genome bisulfite sequencing (WGBS) data, as well as methylation group (HM450Chip) and transcriptome (RNA-seq or expression array) data obtained from the GEO website. Diverse datasets exhibit distinct data processing workflows for mitochondrial DNA and its transcripts. Unless specifically targeted in studies pertaining to mitochondria, these data are likely to be regarded as "background" or "interference" and consequently filtered out. Furthermore, due to the preliminary exploratory nature of this study and the limited sample size, the methylation changes of genes significantly associated with mitochondrial dysfunction in cfDNA failed to reach the biologically meaningful threshold we established, resulting in their being overshadowed by other statistically significant signals. In summary, due to the complexity of the pathogenesis and the heterogeneity of individual manifestations in SLE, further exploration of the critical role of mitochondrial dysfunction in the pathogenesis of SLE is warranted. This necessitates the initiation of larger-scale and more targeted research.

Our study also has some limitations. First, we had limited access to real-world independent validation sets to verify the diagnostic efficiency of cfDNA methylation-related models in patients with SLE. Our previous studies have preliminarily confirmed that the concentration of cfDNA in active SLE patients is significantly higher than that in non-active patients (14). However, in this study, due to funding constraints, we only performed whole-genome bisulfite sequencing (WGBS) on a limited number of samples, which can lead to significant differences in disease activity between samples. Consequently, we did not proceed with an analysis of the relationship between patients' anti-dsDNA antibody levels and cfDNA methylation levels, as the smaller sample size was insufficient to ensure the statistical power for such a correlation analysis. Subsequently, the integration of clinical parameters and cfDNA methylation analysis through a sizable cohort of patients will be a pivotal research objective for us. Secondly, Oxidative stress-induced demethylation of DNA promotes the occurrence of lupus (51), but the underlying molecular mechanisms remain unclear. Unfortunately, we are currently unable to locate data on oxidative stress biomarkers related to the demethylation mechanism of SLE. Utilizing the same batch of plasma cfDNA, quantitative analysis of DNA oxidative damage markers is conducted via Liquid chromatography-mass spectrometry (LC-MS) technology (52). In conjunction with WGBS data, this approach facilitates the exploration of the impact of oxidative damage on the epigenome. Unfortunately, given constraints such as limited sample sizes and insufficient funding, we currently lack the resources to further pursue the experimentation associated with this research.

## 5 Conclusion

We analyzed the immune microenvironment of renal tissue and blood, including plasma cfDNA, in patients with SLE at a multi-group level. The diagnostic model based on plasma cfDNA methylation level has good diagnostic efficiency. A group of 10 DMGs were identified as potential biomarkers for clinical application in patients with LN.

## Data Availability

The datasets presented in this study can be found in online repositories. The names of the repository/repositories and accession number(s) can be found in the article/**Supplementary Material**.
