# Supplementary material for: Plasma cell-free DNA genome-wide methylation profiling enables detection and activity assessment in systemic lupus erythematosus
Source: Front Immunol. 2025 Dec 19;16:1721954. doi: 10.3389/fimmu.2025.1721954 (PMC12757368; doi:10.3389/fimmu.2025.1721954)
Supplement: Supplementary file 1 [file DataSheet1.docx]

Supplementary Material

## Supplementary Figures


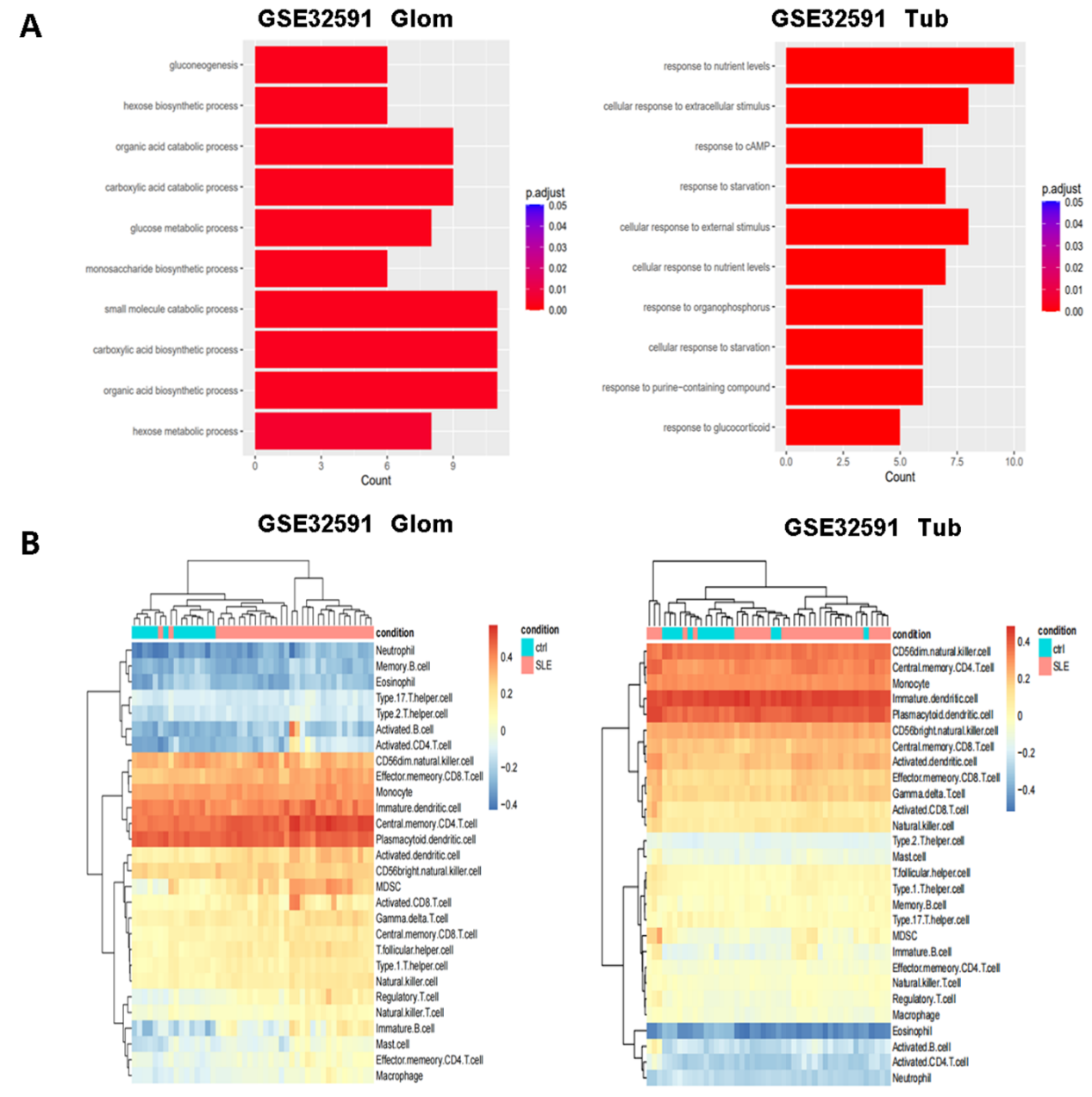


**Figure S1. Enrichment analysis of the glomeruli and renal tubules in patients with LN.**

A. GO analysis (Biological process) of downregulated gene expression in the glomeruli (left) and renal tubules (right) of patients with LN; B. ssGSEA of the glomeruli (left) and renal tubules (right) in patients with LN.


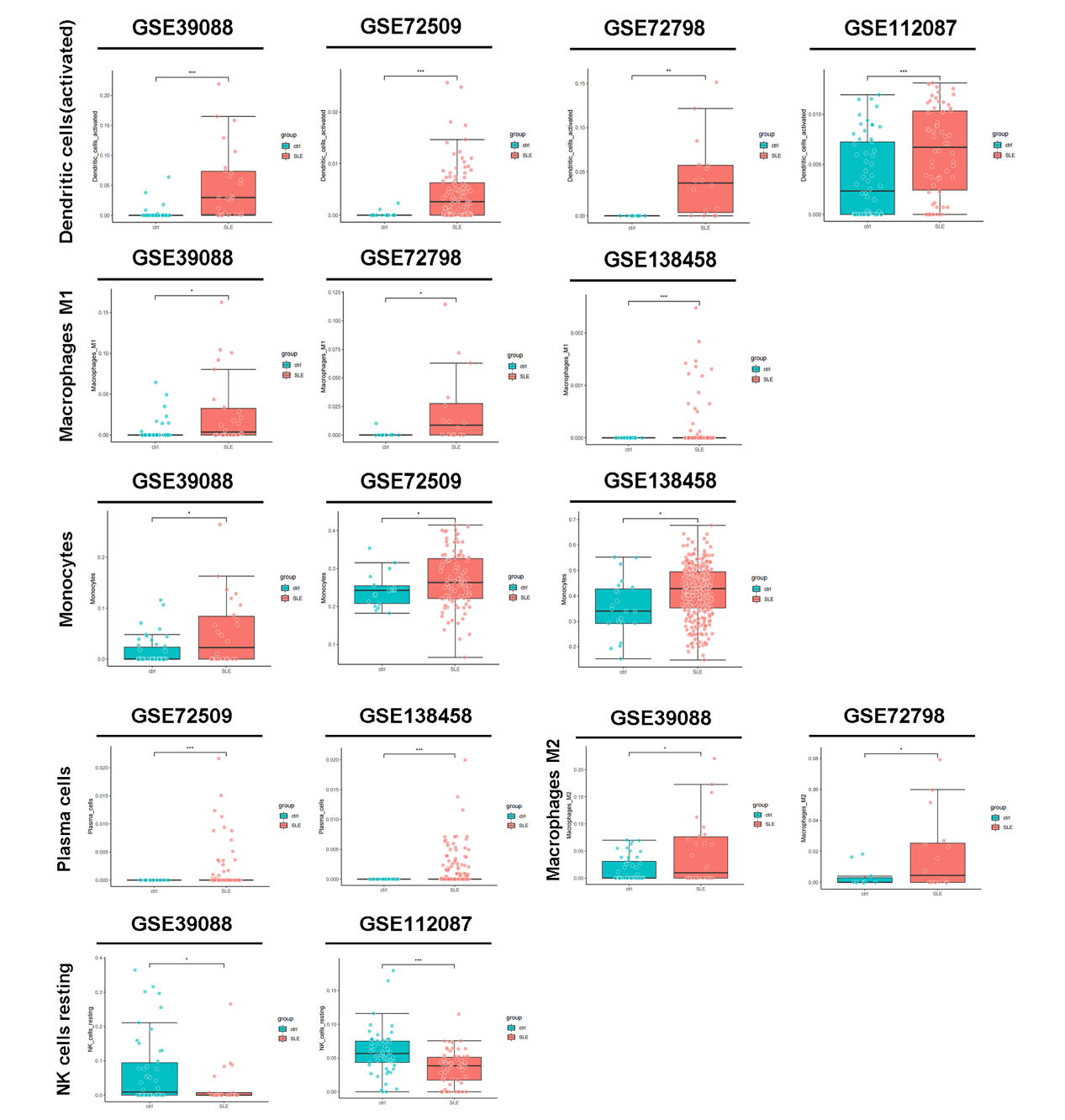


**Figure S2. Immuno-infiltration analysis of whole blood transcriptome in patients with SLE.**


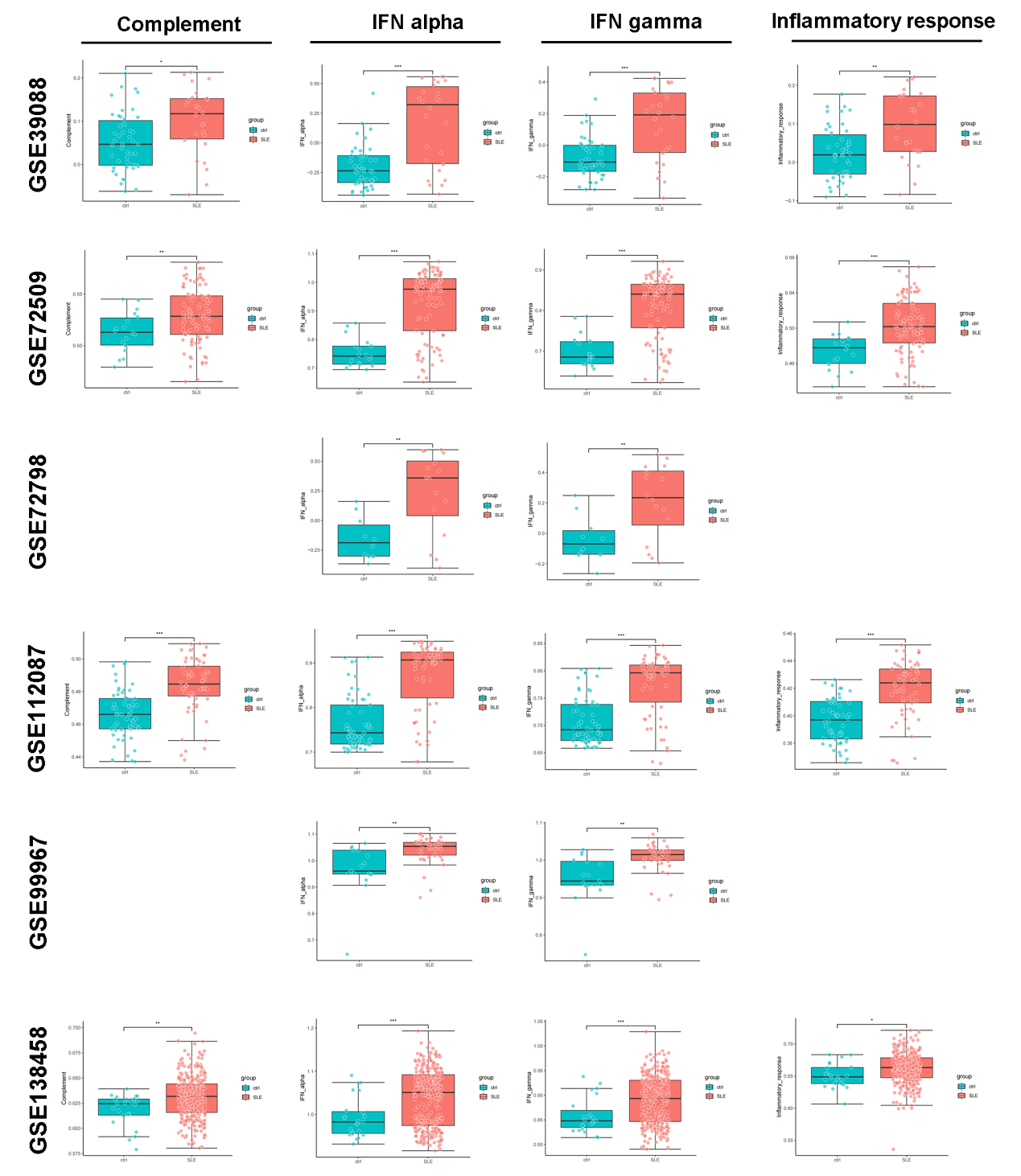


**Figure S3. Whole blood transcriptome immune-microenvironment analysis in patients with SLE.**


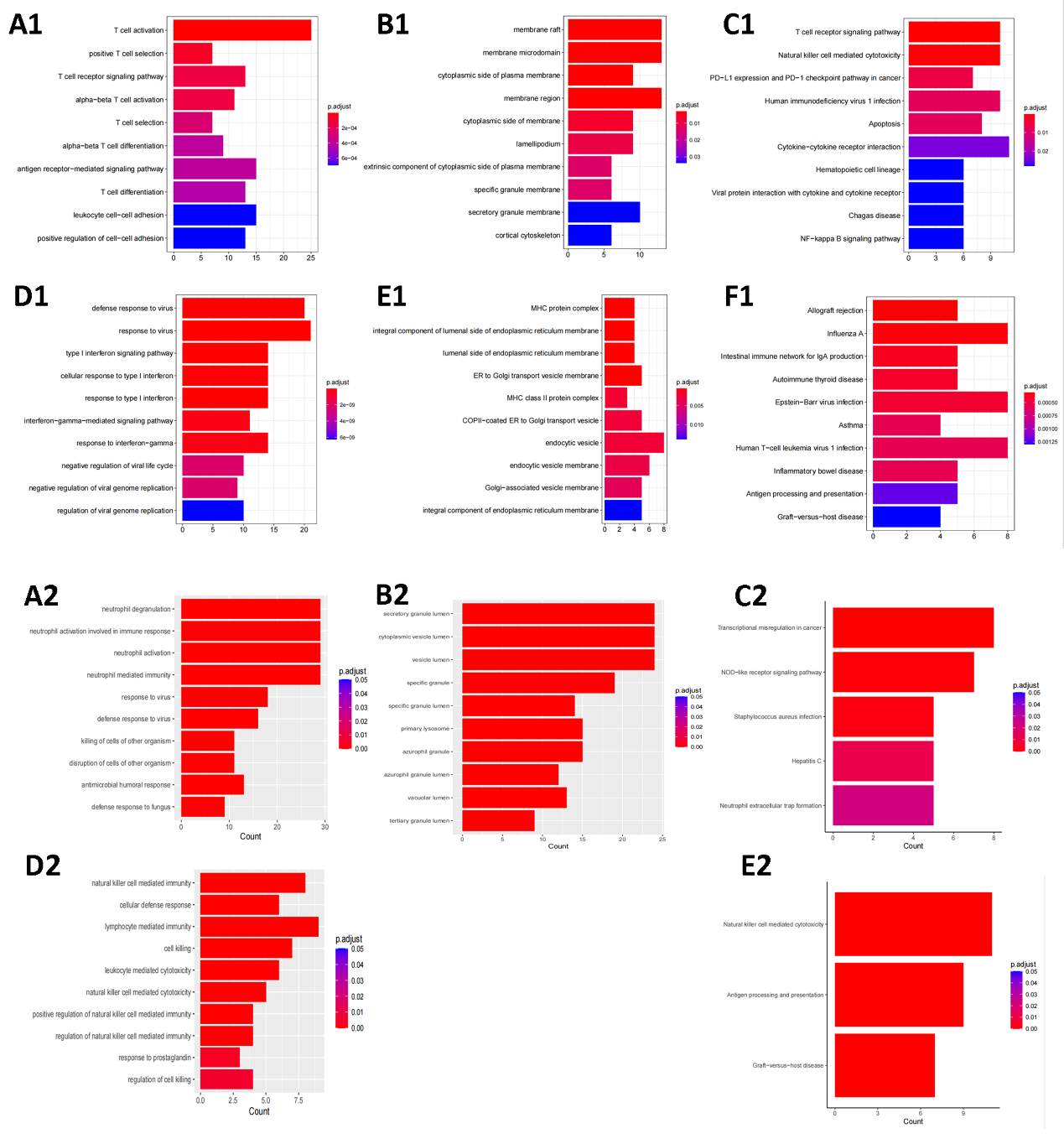


**Figure S4. DMGs and DEGs enrichment of PBMC in the peripheral blood of patients with SLE in the same cohort.**

A1. GO analysis of hypermethylated genes in DMGs from the GSE82218 dataset (Biological processes); B1. GO analysis of hypermethylated genes in DMGs from the GSE82218 dataset (cellular component); C1. KEGG analysis of hypermethylated genes in DMGs in the GSE82218 dataset; D1. GO analysis of hypomethylated genes in DMGs in the GSE82218 dataset (Biological processes); E1. GO analysis of hypomethylated genes in DMGs in the GSE82218 dataset (cellular component); F1. KEGG analysis of hypomethylated genes in DMGs in the GSE82218 dataset; A2. GO Analysis of upregulated genes in DEGs dataset GSE81622 (Biological Processes); B2. GO analysis of upregulated genes in DEGs dataset GSE81622 (cellular component); C2. KEGG analysis of upregulated genes in DEGs in the GSE81622 dataset; D2. GO analysis of downregulated genes in DEGs dataset GSE81622 (Biological Processes); E2. KEGG analysis of downregulated genes in DEGs in the GSE81622 dataset.


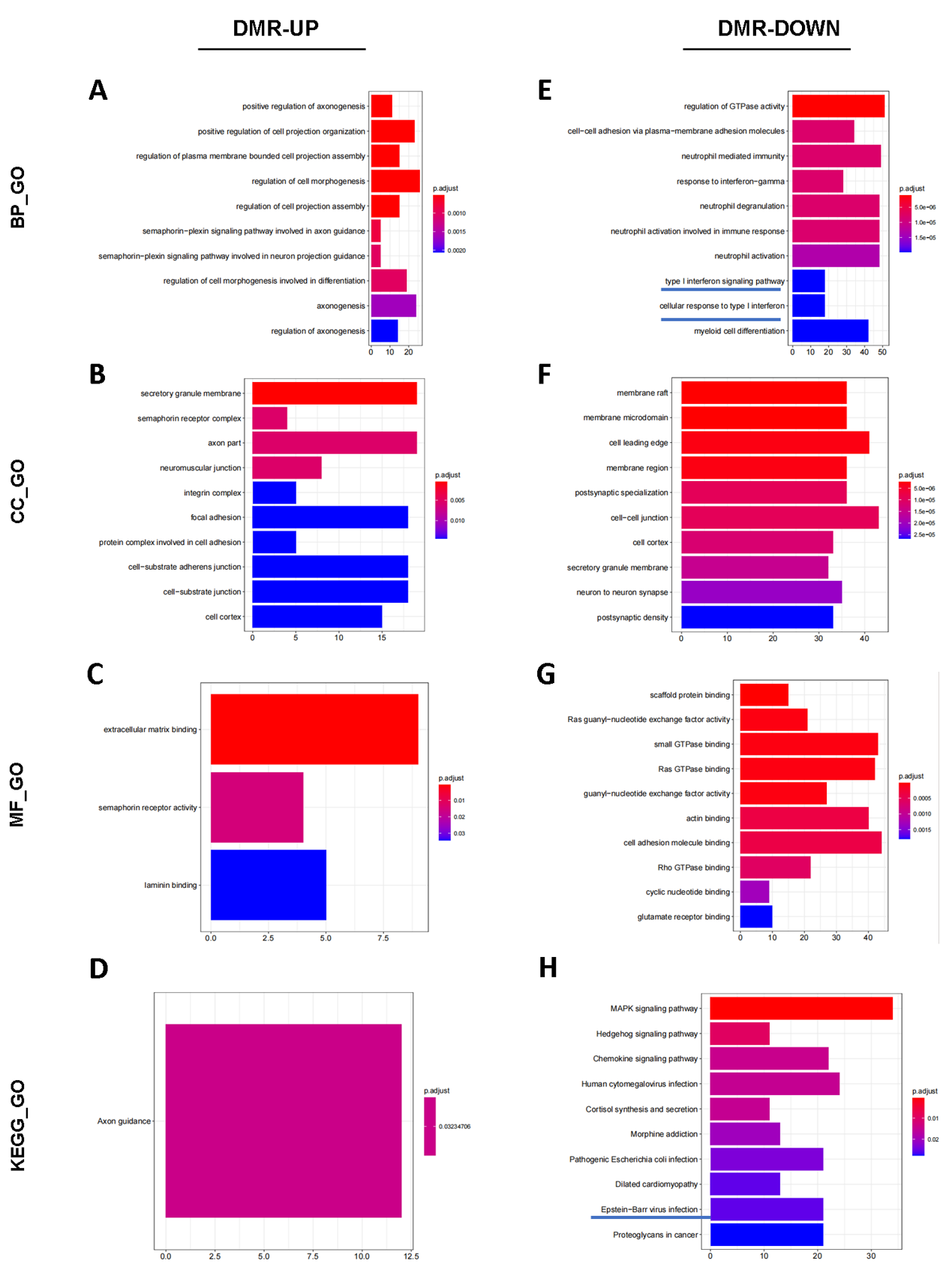


**Figure S5. Enrichment analysis of cfDNA differential methylation region in the plasma of patients with LN.**


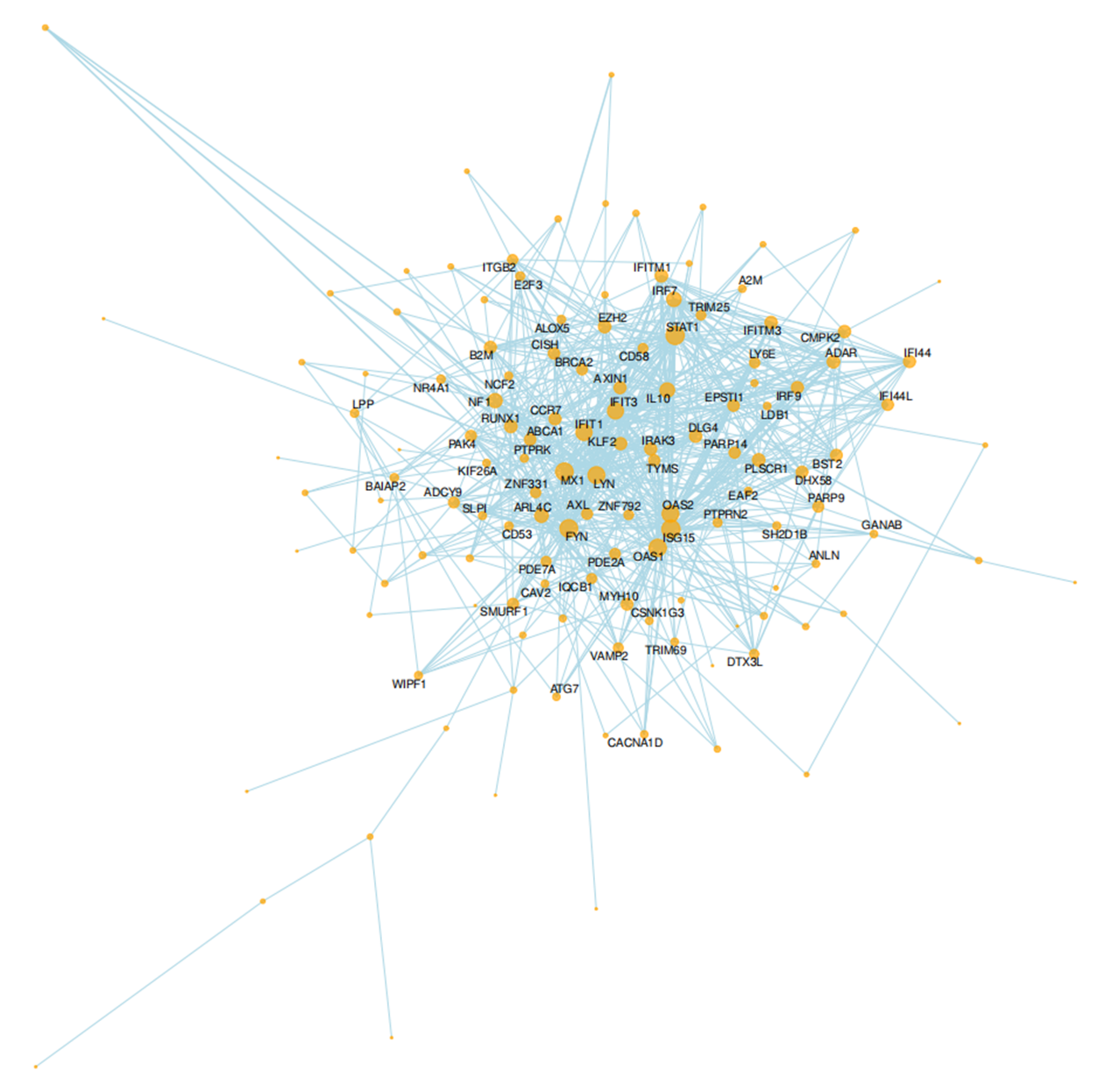


**Figure S6 Protein interaction network constructed with model gene**
